# An Animal Model of Modic Changes by Embedding Autogenous Nucleus Pulposus inside Subchondral Bone of Lumbar Vertebrae

**DOI:** 10.1038/srep35102

**Published:** 2016-10-07

**Authors:** Chao Han, Tao Wang, Hong-qiang Jiang, Jian-xiong Ma, Peng Tian, Jia-cheng Zang, Xin-long Ma

**Affiliations:** 1 Tianjin Hospital, No. 406 Jiefangnan Road, Hexi District, Tianjin City 300211, PR China

## Abstract

The establishment of Modic changes (MCs) in animal model was vital for research of MCs. Fifty-four rabbits were divided into a sham group, a muscle embedment group (ME group) and nucleus pulposus (NP) embedment group (NPE group). In the NPE group, the discs were exposed by the lumbar anterolateral surgical approach. A needle was used to puncture the L5 vertebral body close to the endplate. NP was extracted by a syringe from L1/2 intervertebral discs and then injected into the drilled hole of subchondral bone. The muscle embedment group and sham group had the same procedure and drill method as the NP embedment group. Some pieces of muscle were put into the hole in the ME group, but nothing was put into the hole in the sham group. After the operation, MRI scan and molecular biology tests were applied. The signal changes were found in the NPE group; while the sham group and the ME group showed no significant signal change. Histological observation confirmed that there was abnormal tissue proliferation in imbed site. High expression of IL-4, IL-17 and IFN-γ were detected in the NPE group. The embedment of NP into subchondral bone can create an animal model of MCs.

Modic changes (MCs) are vertebral endplate and adjacent bone marrow lesions visible via magnetic resonance imaging (MRI). They are quite common in individuals who had the relative symptoms^1^. The importance of MCs has been highlighted in many studies due to their association with lower back pain (LBP)^2^,^3^. Three different types of subchondral signal abnormalities in vertebral body marrow were first described independently by de Roos *et al*.^4^. and Modic *et al*.^5^. Modic I type changes reflect a hypointense signal in T1-weighted (T1W) and hyperintense signal in T2-weighted (T2W) sequences. Fissured endplates with adjacent vascular granulation tissue within the bone marrow were found in such lesions. Modic II type changes show a hyperintense signal in both T1W and T2W sequences. Disruption of the endplates as well as histological fatty replacement of the adjacent bone marrow could be detected in this type of lesion. Modic III type changes reveal a hypointense signal in T1W and T2W sequences. Lesions with sclerotic corresponding to the endplate were observed^6^. MCs have been considered to be a pathological spinal condition that is closely related to many degenerative diseases in the spine^7^,^8^,^9^.

To review the current evidence, some studies have already focused on the detailed knowledge of aetiology and pathomechanism of MCs. Albert *et al*. hypothesised that MCs were possibly induced by intervertebral disc herniation^8^ Hu *et al*. ascribed MCs to severe intervertebral disc degeneration^10^. Crock brought up the concept of “internal disc disruption” where repeated trauma to the disc could result in micro fissures on the endplate. Once a fissure formed, the breach of the endplate by the nucleus pulposus (NP) may induce an autoimmune reaction and further cause MCs^11^. Ma *et al*. held the similar opinion and reported that autoimmunity raised by NP played a key role in the pathogenesis of MCs^12^.

Immune system cells, especially CD4^+^ T helper lymphocytes, play a critical role in the pathogenesis of autoimmunity^13^. The recently identified Th17 subset producing proinflammatory cytokine IL-17 promotes chemokine expression and stimulates T cells producing IFN-γ in the damaged organs^14^. Th2 cells also have a distinct role in the pathogenesis of immune reaction. IL-4 as the representative Th2 cell expression may lead to severe immunopathological consequences^15^.

Although there were many studies regarding MCs in clinical settings^16^,^17^,^18^,^19^,^20^,^21^,^22^,^23^,^24^, appropriate experimental animal models that mimic the MC process often occurring in humans and that can be used for etiological studies or new treatment such as target therapy are lacking. To date, only a few animal models of MCs have been reported to investigate potential pathological mechanisms.

On the basis of autoimmunity theory proposed by Albert and Ma, the aim of this study was to establish a simple, reproducible rabbit model of MCs by the autotransplantation of NP into the drilled vertebral body next to the endplate. Other goals were to observe the characteristics of animal models in histology and to evaluate the specific mechanism of NP in the development of MCs. To achieve these aims, methods such as molecular biology, MRI, and histologic studies were performed to investigate the progress of MCs.

## Results

### General Conditions of Animals

Two rabbits died during surgery as a result of bleeding and four rabbits died in the process of the anesthesia during the MRI scan. The remaining 48 rabbits survived with no postoperative behavioral or neurologic symptoms.

### MRI Assessment

**Figure 1:**
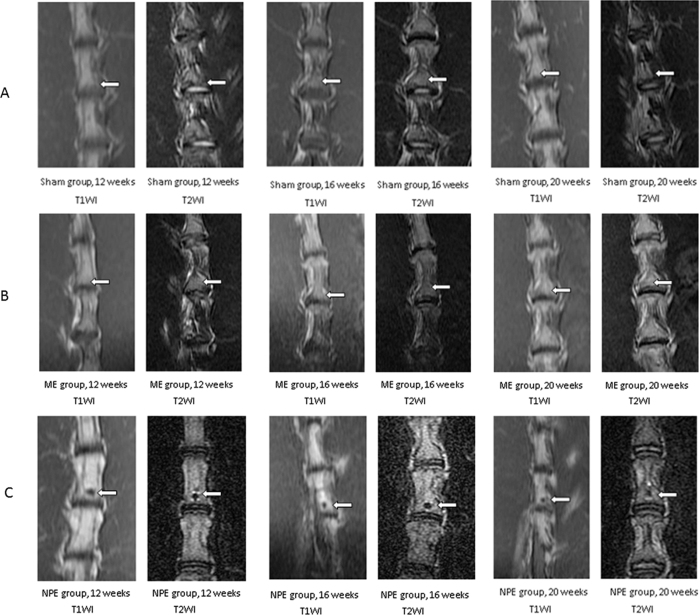
(**A**) Representative serial MRI scans of the lumbar spine of a rabbit in three time points. In the sham group, no signal abnormality was observed in the picture. (**B**) The characteristic of vertebral body signal in the ME group was similar to the sham group. As time elapsed, there was no significant signal change in the embedment position. (**C**) In the NPE group, the hypointense signal in T1W and mixed signals with hypointense in T2W sequences were easily observed. From the 12-week time point to the 20-week time point, the sporadic hyperintense signals around the hypointense signal in T2W were diminished.

MRI showed a different degree of signal intensity depending on the different tissues embedded in the drilled holes. At the 12-week, 16-week, and 20-week time points after embedment of the L5 vertebral body, progressive changes of signal intensity (hypointense signal in T1W and mixed signals with hypointense in T2W sequences) were apparent for NPE group (Fig. 1C). In contrast, MRI appearances of the embedment position of the other two groups remained relatively constant over the same period (Fig. 1A,B).

### Gross Anatomy Observation

**Figure 2:**
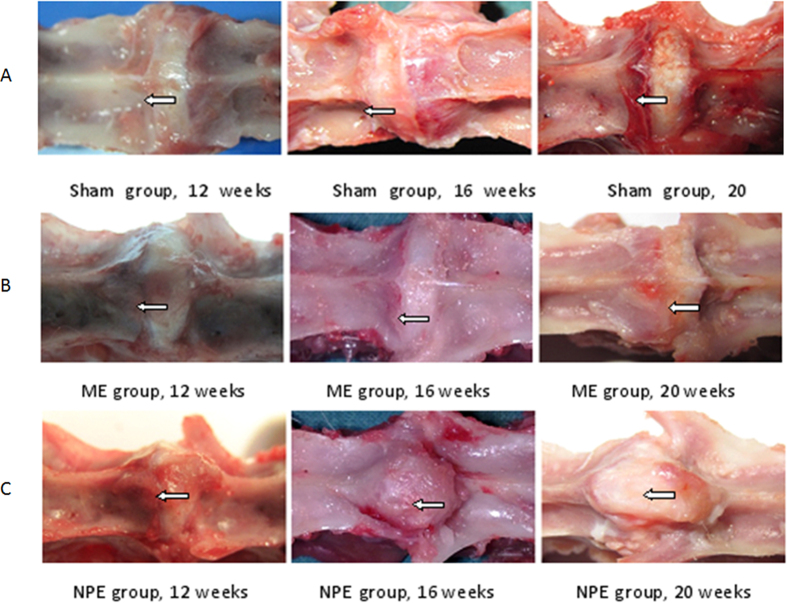
(**A**) The surface of the vertebral body in the embedment position was very smooth; the drilled hole healed well. No proliferation was observed in the vertebral body. (**B**) The characteristic of the embedment position in the ME group was similar to the sham group. As time elapsed, there was no obvious appearance change in the embedment position. (**C**) The hyperostosis in the embedment position was formed in the NPE group. The hyperostosis increased rapidly, and even came across the disc to the contralateral vertebral body.

In the group of NPE, an obvious hyperostosis could be found in the NP embedment position. From 12-week to the 20-week time point, the proliferation of bone was very quick (Fig. 2C). Compared to the NPE group, there was no distinct change of modeling vertebral body in both the sham group and the ME group (Fig. 2A,B).

### Histological Evaluation

**Figure 3:**
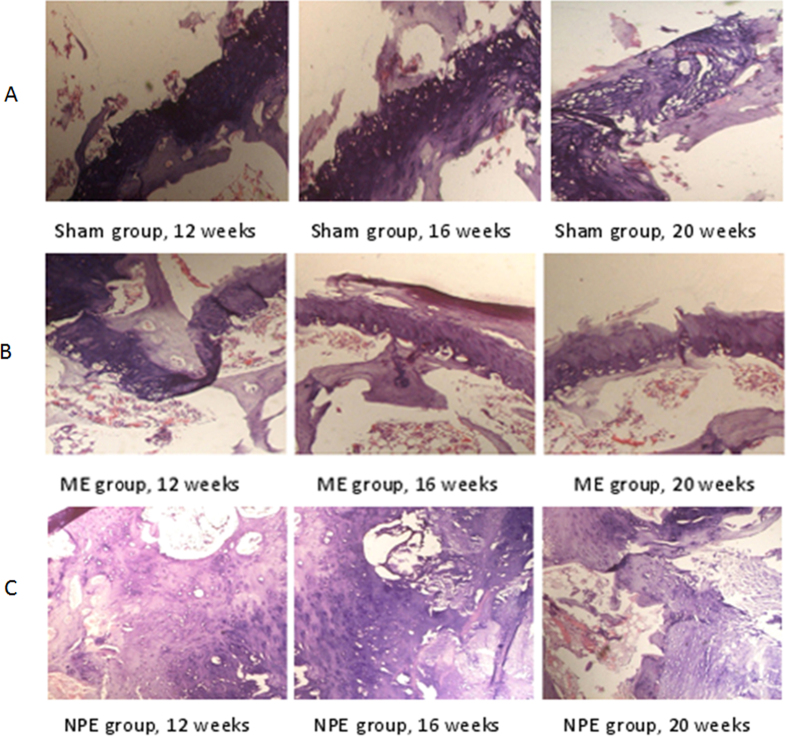
(**A**) The trabecula could be seen adjacent to the endplate. Cartilage cells were arranged in neat rows, the dimension and shape of the cells were homogeneous, and no proliferation was observed (40×). (**B**) The condition of the embedment position in the ME group was similar to the sham group. Both trabecula and chondrocyte could be seen, and there was no apparent proliferation in the embedment position (40×). (**C**) The apparent proliferations of cartilage cells and NP-like cells could be observed. The shape and dimension of the cartilage cells were not of uniform size (40×).

Histological analysis was performed to provide more details on the bone formation. Figure 3 shows the photographs of H&E staining sections after surgery. In the sham group, the chondrocyte was well arranged and no proliferation of cells could be found (Fig. 3A). The condition of ME group was similar to the sham group (Fig. 3B). However, in the NPE group, a significant amount of chondrocyte and NP-like cell proliferation were observed in the embedment position; trabecular structure was hardly observed in the picture (Fig. 3C).

### RT-qPCR Analysis

**Figure 4:**
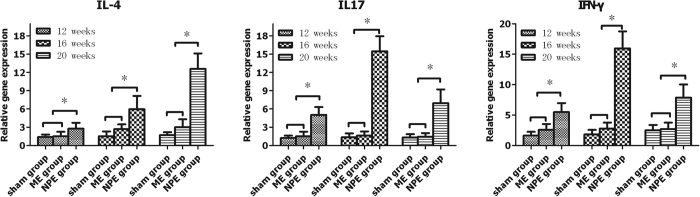
The mRNA expressions of IL-4, IL-17, and IFN-γ in the NPE group tended to significantly rise compared to the sham and ME groups (P < 0.05). On the contrary, the expression level of the ME group did not show significant differences (P > 0.05).

Interleukin-4 (IL-4) mRNA, interleukin-17 (IL-17) mRNA, and interferon-γ (IFN-γ) mRNA were expressed in both the NPE group and the ME group. When we compared the expression levels of the target genes, a statistically significant increase in the gene expression of IL-4, IL-17, and IFN-γ was seen in the NPE group compared with the ME group and the sham group (Fig. 4) (P < 0.05). When we compared the ME group to the sham group, the expression levels of IL-4, IL-17, and IFN-γ were just slightly increased and did not reach a statistically significant change (P > 0.05).

### Western Blotting Analysis

**Figure 5:**
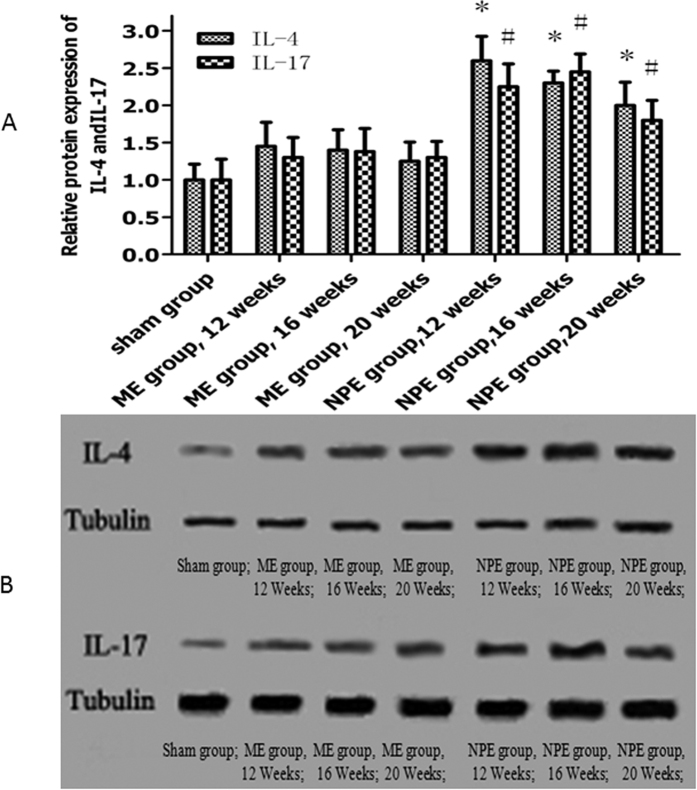
(**A**) The protein levels of IL-4, IL-17 were significantly increased in the NPE group compared with the ME group and the sham group (P < 0.05). (**B**) Western blot strip chart.

IL-4 and IL-17 were subjected to western blot analysis with commercially available antibodies to confirm the modified mRNA expression patterns. As shown in Fig. 5A,B, a statistically significant increase in the protein levels of IL-4 and IL-17 was noticed in the NPE group compared with the ME group and the sham group (P < 0.05). Compared to the sham group, the protein levels of IL-4 and IL-17 in the ME group also did not reach a statistically significant change (P > 0.05).

## Discussion

Because of limited access to human samples from the surgery, definitive and detailed studies of the pathogenesis of MCs have been somewhat obstructed. We have performed the attempt of establishment of MC animal models in order to investigate their potential pathological mechanisms. At the same time, radiographic assessment, histologic assessment, and molecular biology assessment were applied to follow the process of MCs caused by autotransplantation of NP. As a result, the NP embedment model led to a progressive change of signal intensity (hypointense signal in T1W and mixed signals with hypointense in T2W sequences) from the 12-week to the 20-week time point, indicating the changes of the tissue with histologic and molecular biology assessment supporting radiologic results.

The results of this experiment indicated that imaging and histological changes have emerged in the embedment position of the NPE group. At the same time, we detected a statistically significant increase in the gene expression of IL-4, IL-17, and IFN-γ and the high-level protein expression of IL-4 and IL-17 in the embedment position of the NPE group. Those changes indicated that the embedment of autologous NP in the vertebral body could induce a series of signal and morphological changes. It was not difficult to find that the signal characteristic (hypointense signal in T1W and mixed signals with hypointense in T2W sequences) of an animal model is quite similar to the MCs of humans. Also, the MRI finding supported the histologic and gross anatomy observation evidence that the change of MCs is progressive. Although the inflammatory response caused by acute injury may be turned out shortly after puncture, MRI results show that the gradual signal changes had been present at 12 weeks after puncture, and continuing through 20 weeks, with no sign of any recovery or reversal of MRI changes. These results suggest that autologous NP in the vertebral body is a reliable method for building a progressive form of MCs in the rabbit.

This puncture model requires adequate skill, time, and cost of surgery. In the preliminary experiments, the peeling or excessive stimulation of paraspinal ligamentous structures was likely to result in the formation of vertebral osteophytes. Care should be taken to avoid injury or irritation of adjacent discs. Because the controlled depth of puncturing is necessary to create stable and reproducible results, we made a handmade stopper by cutting a length of 3 mm of needle cover. Using this stopper, the same drill depth through the vertebral body can be ensured. In the preliminary experiments, the three orthopaedic surgeons involved in the operation procedure considered that the 16-gauge needle was easier to operate than the 18-gauge needle or other modes. In order to avoid the intense bleeding from the drilled hole, holding the needle still for a while will result in a more suitable hole for embedding, suggesting the possibility to control a degree of MCs by this means.

Although many studies were aimed at research on MCs, the aetiology and pathomechanism of MCs have been poorly understood^25^,^26^,^27^. Based on our former research, we found that autoimmunity plays a key role in the process of MCs^12^. In our study, we present the investigation of the quantitative expressions of IL-4, IL-17, and IFN-γ, the cytokines presenting the major pathways of CD4+ cell differentiation after antigenic stimulus. In our study, the relevant highly expressed IL-4, IL-17, and IFN-γ and increased protein levels of IL-4, IL-17 were found in the NPE group compared with the negative groups.

In clinic, an increased IL-17 mRNA expression has been described in NP cells from patients with intervertebral disc herniation^28^. Compared to the healthy controls, increased IL-4 and IFN-γ expression levels have also been found in the model of acute noncompressive disc herniation^29^ IL-17 has a critical role in tissue injury in inflammatory, autoimmune diseases^30^ and enhances the immune responses in IFN-γ^31^. Enhanced IL-17-mediated tissue damage was reported in MRL/lpr mice^32^ and in autoimmunity-prone mice^33^. IL-4 has the ability to inhibit the expression of pro-inflammatory cytokines such as IL-1β and TNFα, as well as the activation of macrophages^34^. Variable expressions of IL-4 mRNAs have been reported in the groups of NPE; its rising tendency was in contrast with IL-17 and IFN-γ at the same time point. The mRNA expression of IFN-γ in the NPE group was significantly greater than in other groups. Therefore, IFN-γ production may be a mediator of an inflammatory response induced by the embedment of NP. It has been shown that IFN-γ is produced by multiple cell types including activated type 1 helper T-cells, natural killer cells, and macrophages^35^,^36^, and is a key proinflammatory cytokine that can contribute immune response^37^.

Our work indicates that autoimmune response is likely to be involved in the occurrence of MCs and their development process. Luoma *et al*. noticed that the signal characteristic between MCs and herniated NP was similar in MRI; both presented hyperintense signal in T2W sequences^38^. Some cytokines, such as IL-1, have been confirmed that is closely related with the occurrence of MCs^39^. Ma *et al*. believed that the upward or downward herniation of NP may exert a great effect on the development of MCs^12^. Bobechko^40^ and Gertzbein *et al*.^41^. reported that the NP was an immunologically tolerant tissue that was not accessible to the vascular circulation from birth. Herniation of the NP introduced a foreign body into the blood supply, which mediated an autoimmune response in the local position^42^. Autoimmune reaction could induce the immune factors in large quantities, and when those factors worked on the organisation constantly, they would have the potential to induce the signal changes^43^. In the study, the overexpressed IL-4, IL-17, and IFN-γ were typical immune factors; this further showed the close relationship between NP and MCs^44^. Our experimental animal model well simulated the process of NP breaking through the endplate into the vertebral body and further revealed the effect of autoimmunity on MCs.

As expected, this animal model provides us with a possible platform to investigate the MCs. However, there are still some limitations on this model: first, during the animal observation phase, it was necessary to euthanise some rabbits in the middle of stages for the histology and molecular biology tests, so some animals were “dropped out” as time progressed. Second, although three time points were set in this study, unfortunately, we simulated just one type of MC (Modic I type changes), so it is inadequate to represent the development process of human disease and more time points should be set to better observe the whole signal changes. Third, it is true that the change of tissue structures could be clearly demonstrated by histological staining, but some specialised techniques may better reveal the change of microstructure in the present model. For instance, polarised light microscopy was used to analyse the formation of fibrocartilage in rabbit intervertebral discs^45^. The long-term effects of NP on MCs and endplates require further study.

## Material and Methods

### Animals

A total of 54 male New Zealand White rabbits (weighing approximately 2.5–3 kg each and ranging in age from 3 to 3.5 months old) were randomly divided into three groups: a sham group, a muscle embedment group (ME group), and an NP embedment group (NPE group). All of the experimental procedures were approved by the Ethics Committee of Tianjin Hospital. The methods were carried out in accordance with the approved guidelines.

### Surgical Procedure

**Figure 6:**
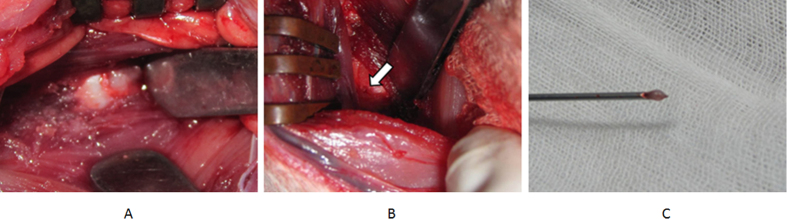
(**A**) The exposure of L5–L6 disc by posterolateral retroperitoneal approach. (**B**) The hole was drilled by a 16-gauge needle in the L5 vertebral body adjacent to endplate. (**C**) The harvested autologous NP.

The S. Sobajima surgical method^46^ was performed with some modifications. Each rabbit was placed into a lateral prone position and a posterolateral retroperitoneal approach was used to expose the anterior surfaces of five consecutive lumbar intervertebral disc (IVDs). Each rabbit was put under general anesthesia (injection with 20% ethyl carbamate via ear vein, 5 ml/kg). A longitudinal skin incision was made from the inferior margin of the rib cage to the pelvic rim, 2 cm ventral to the paraspinal musculature. The right anterolateral vertebral column from L1 to L6 was exposed by sharp and blunt dissection of the overlying subcutaneous tissue, retroperitoneal fat, and musculature (Fig. 6A). Disc levels were identified using the pelvic rim as an anatomic landmark for the L5–L6 disc level. The L5 vertebral body adjacent to endplate was drilled by a 16-gauge needle to a depth of 3 mm (Fig. 6B). The autologous NP was harvested by the aspiration of a syringe (5 ml) in the L1–L2 disc (Fig. 6C). According to the requirements of each group, the NP or muscle was pushed into the drilled hole. After drill and embedment, the deep fascia, superficial fascia, and skin were closed in layers with absorbable sutures. Throughout all procedures, care was taken to not disturb the periosteal tissues of the vertebrae.

Under general anesthesia by the injection with 20% ethyl carbamate via ear vein (5 ml/kg), radiographs of the lumbar spine were repeated at 12, 16, and 20 weeks after surgery.

The rabbits were killed at 12, 16, and 20 weeks post surgery by intramuscular injection of ketamine (25.0 mg/kg) followed by an intravenous injection of sodium pentobarbital (1.2 g/kg), and the intact spinal columns were harvested for histologic analysis. Quantitative real-time reverse transcription-polymerase chain reaction (RT-qPCR) and western blot analysis were also applied to measure the changes of immune factors.

### Magnetic Resonance Imaging

MRI examinations performed on the rabbits were obtained using a 3.0 T clinical magnet (General Electric Medical Systems, Florence, SC) with a quadrature extremity coil receiver. Rabbits were anaesthetised by injection with 20% ethyl carbamate via the ear vein (5 ml/kg) and placed supine within the magnet, with the lumbar region centred over a 5-inch diameter circular surface coil (General Electric Medical Systems). A coronal T2-weighted localiser image (TR, 1445 ms; TE, 37 ms) was obtained to establish the position of the lumbar discs from L3–L4 to L5–L6. T2-weighted sections in the sagittal plane were obtained in the following settings: fast spin echo sequence with time to repetition (TR) of 2200 ms and time to echo (TE) of 70 ms; 256 (h) × 128 (v) matrix; field of view of 260; and eight excitations. The section thickness was 2 mm with a 0.2-mm gap.

### Histologic Analysis

After the final radiograph was obtained and the last rabbit was killed, sham, muscle embedment, and NP embedment discs were harvested for histologic studies. The tissues were fixed in 10% neutral buffered formalin for 1 week, decalcified in ethylenediaminetetraacetic acid, and processed for paraffin sectioning. Blocks of tissue were embedded in paraffin and cut into sagittal sections (5 μm in thickness) using a microtome. The sections were stained with haematoxylin and eosin (H&E).

### Extraction of Total RNA and Reverse Transcription to cDNA

After harvesting the discs from rabbits in each group, total RNA was extracted using UNIQ-10 column (Sangon Biotech Co. Ltd., Shanghai, China) according to the manufacturer’s instructions and reverse-transcribed using ImProm II Reverse Transcription System (Promega Corporation, Madison, WI, USA).

### RT-qPCR with SYBR Green

RT-qPCR was performed using a Prism 7300 (Applied Biosystems Inc., USA) and the SYBR Green Jump Start Taq ReadyMix (Sigma-Aldrich, St. Louis, MO, USA) according to the manufacturer’s specifications. The PCR reaction volume was 20 μL containing 1.5 μL of diluted cDNA and 0.2 μM of each primer. Primers were designed by OligoPerfect Designer (Invitrogen, Valencia, CA) and produced by Nanjing Jin Stewart Biological Technology Co., Ltd., China (Table 1).The following thermocycling conditions were employed: initial polymerase activation step for 2 min at 94 °C, followed by 40 cycles of 15 sec at 94 °C for template denaturation, 1 min at 60 °C for annealing, and 1 min at 72 °C for extension and fluorescence measure. All samples were amplified in triplicates and the mean was used for RT-qPCR analysis. Amplification data were analysed using FlexStation 3 (Molecular Devices, Sunnyvale, CA, USA). The expressions of the IL-4, IL-17, and IFN-γ genes were normalised to that of the endogenous control (ACTB). Relative levels of target mRNA expression were calculated using the 2^−ΔΔ^CT method.

### Western Blotting Analysis

Total protein was extracted from the tissue in RIPA lysis buffer (containing protease and phosphatase inhibitor mixtures) by using a tissue homogeniser, followed by clearing tissue debris by centrifugation at 13000 rpm at 4 °C for 20 min. Fifty micrograms of protein were loaded per lane and separated by 10% SDS-PAGE gel electrophoresis, then transferred onto PVDF membranes. Blocking was carried out in 5% nonfat dry milk in Tris-buffered saline (TBS) containing 0.1% Tween 20 for 1 h at room temperature. The membranes were incubated with primary antibody rabbit antidecorin (diluted 1:200; Boster, Wuhan, China); anti-biglycan (diluted 1:200; Bioss, Beijing, China) overnight at 4 °C and with secondary antibody (1:40000 dilution of goat antirabbit immunoglobulin G) conjugated to horseradish peroxidase (Boster, Wuhan, China) for 1 h at room temperature on the following day. Immunoblotting signal was detected by enhanced chemiluminescence on chemiluminescent films following exposure to an X-ray. For densitometric analyses, the blots were scanned and quantified using BandScan software, and the result was expressed as the ratio of target gene immunoreactivity to tubulin immunoreactivity.

### Statistical Analysis

Statistical calculations were performed using software kit SPSS16.0 (SPSS, USA). The data collected from study were expressed as mean ± standard deviation (mean ± SD) and analysed by one-way repeated measures analysis of variance (ANOVA) to determine differences between two groups. P < 0.05 was considered statistically significant.

## Conclusions

In summary, the establishment of an MCs animal model by autologous NP embedment into the vertebral body, along with gross anatomy observation, MRI analysis, histologic evaluation, and molecular biology analysis, may prove to be a significant tool in the assessment and understanding of the mechanism of MCs as seen in humans, as well as in the development of new therapeutic interventions.

## Additional Information

**How to cite this article**: Han, C. *et al*. An Animal Model of Modic Changes by Embedding Autogenous Nucleus Pulposus inside Subchondral Bone of Lumbar Vertebrae. *Sci. Rep*. **6**, 35102; doi: 10.1038/srep35102 (2016).

## Figures and Tables

**Table 1:** The Primer Sequences.

Gene	Primer	Sequence(5′-3′)
IL-4	Primer F	CACTCTGCTCTGCCTCCTC
	Primer R	CTCTCCGTGCTCCTTGAAG
IL-17	Primer F	CGAGAGGAACCTTGGGGAG
	Primer R	CACGGGAACCTGGCTGAAC
IFN-γ	Primer F	GCCAAATTGTCTCCTTCTAC
	Primer R	CTGACTCCTTTTTCGCTTC
ACTB	Primer F	ATGCAGAAGGAGATCACC
	Primer R	AACACGAATAAAGCCATG
